# Induced nano-scale self-formed metal-oxide interlayer in amorphous silicon tin oxide thin film transistors

**DOI:** 10.1038/s41598-018-22602-4

**Published:** 2018-03-07

**Authors:** Xianzhe Liu, Hua Xu, Honglong Ning, Kuankuan Lu, Hongke Zhang, Xiaochen Zhang, Rihui Yao, Zhiqiang Fang, Xubing Lu, Junbiao Peng

**Affiliations:** 1 Institute of Polymer Optoelectronic Materials and Devices, State Key Laboratory of Luminescent Materials and Devices, Department of Materials Science and Engineering School, South China University of Technology, Guangzhou, 510640 China; 2 Institute for Advanced Materials and Guangdong Provincial Key Laboratory of Quantum Engineering and Quantum Materials, South China Normal University, Guangzhou, 510006 China

## Abstract

Amorphous Silicon-Tin-Oxide thin film transistors (a-STO TFTs) with Mo source/drain electrodes were fabricated. The introduction of a ~8 nm MoO_x_ interlayer between Mo electrodes and a-STO improved the electron injection in a-STO TFT. Mo adjacent to the a-STO semiconductor mainly gets oxygen atoms from the oxygen-rich surface of a-STO film to form MoO_x_ interlayer. The self-formed MoO_x_ interlayer acting as an efficient interface modification layer could conduce to the stepwise internal transport barrier formation while blocking Mo atoms diffuse into a-STO layer, which would contribute to the formation of ohmic contact between Mo and a-STO film. It can effectively improve device performance, reduce cost and save energy for the realization of large-area display with high resolution in future.

## Introduction

Amorphous oxide semiconductors thin film transistors (AOS-TFTs) have been recognized as a promising candidate for the active-matrix liquid-crystal displays (AMLCDs) and active-matrix organic light emitting diodes (AMOLEDs), owning to their tremendous advantages of lower process temperature, high transparency and good uniformity, as comparison to the conventional amorphous silicon (a-Si) and low-temperature polycrystalline silicon (LTPS) TFTs^1–3^. Initially, TFTs based on ZnO, In_2_O_3_ and SnO_2_ have been intensively reported in many periodicals. The crystallization of these semiconducting thin films leads to poor uniformity in large area. To enhance the device performance, the uniformity and the carrier concentration of semiconducting films should be considered. In general, amorphous phase contributes to the uniformity of semiconducting films, which could be achieved by incorporating dopants with different ionic charges and sizes into semiconductors to increase the crystallization temperature of the film. In addition, the carrier concentration could be optimized by rationally controlling oxygen vacancies in semiconducting film^4^. Based on the dopant’s bond-dissociation energy (BDE) with oxygen atoms and Lewis acid strength of elements, it is convenient for us to identify the appropriate dopant^5^. Many amorphous multiple oxide compounds have been exploited as the functional semiconductor layer, such as a-IGZO^6^, a-IZO^7^, a-ZTO^8^ and a-SZTO^9^. So far, TFT with back channel etching (BCE) structure is beneficial to fabricate displays with high resolution. Tin oxide semiconductor is a good candidate for BCE type TFTs due to anti-acid trait, low electrical resistance and high optical transparency^10^. But its drawbacks are excess carrier concentration and crystallization. The incorporation of SiO_2_ is demonstrated as a good dopant in tin-based films^11,12^.

In addition, in term of device performance, the energy-level alignment at the Source/Drain (S/D) electrode/semiconductor, reflected in the contact resistance, is a key factor because it determines the transport carrier injection barrier. The large contact resistance would lead to the resistive-capacitive (RC) delay which gives rise to image distortion and shading effect in large-scale displays with high pixel density. A small energy-level offset formed at the interface between the semiconductors’ charge transport level and the metal’s Fermi level is always favored to form an ohmic contact. Therefore, the oxide semiconducting layers matched with appropriate electrode materials and some means of decreasing contact resistance have been being extensively investigated. For example, an aluminum oxide layer or titanium oxide layer may easily form at the interface between a-IGZO and electrodes, when metal Al or Ti was used as S/D contacted metal^13,14^. These phenomena will cause a negative threshold voltage (V_th_) shift due to the extraction of oxygen out of channel layer, resulting into the increment of oxygen vacancies in oxide semiconductor. AOS-TFTs using high conductive Cu as S/D electrodes are especially difficult to obtain a high-performance device due to the diffusion of Cu atoms into the channel layer. The Cu in semiconducting film acts as an acceptor-like/donor-like trap that decreases/increases the carrier concentration in the channel layer^15–19^. Therefore, a thin barrier layer is generally introduced to block the diffusion of Cu atom into the AOS layer. Previous works have made a significant contribution to improve the contact quality that improves the device performance.

In this paper, we attempted to improve the contact quality of a-STO TFT matched with Mo electrodes. To well demonstrate the S/D contact effects on electrical performance of device, the contact resistance (R_SD_) and the channel resistance (R_ch_) were extracted by the well-known transmission line method (TLM) based on a series of TFTs with different channel lengths. The experimental results showed that a new self-formed interlayer generated between Mo electrodes and semiconductor at pre-annealing temperature of 300 °C contributed to the improvement of the electrical contact quality and the elimination of Mo diffusion.

## Results and Discussion

**Figure 1:**
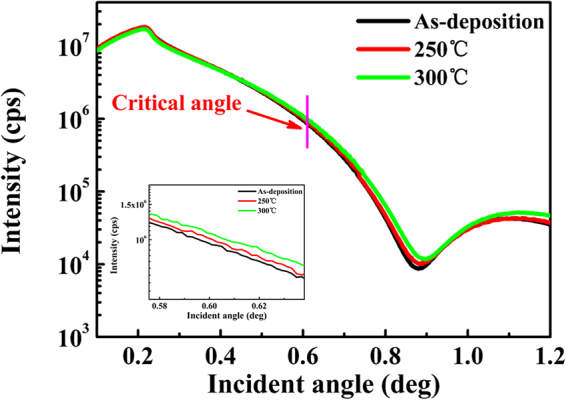
X-ray reflectivity (XRR) curves for a-STO films with different annealing processes (as-deposition, annealed at 250 °C and 300 °C in air ambient). The inset shows the partial magnification in critical angle region.

Figure 1 shows that the X-ray reflectivity (XRR) spectrum of a-STO films. In order to investigate the changes of the film density, thickness, and roughness, the measured XRR spectra were simulated using the X’Pert Reflectivity software. For the XRR measurement, the oscillation is represented by the interference of two reflected X-rays: a beam of X-ray is reflected at the air/film interface, and a beam of X-ray is reflected at the film/substrate interface. As the film thickness increased, the critical angle (θ_c_), the oscillation period and the amplitude are reduced. Simultaneously, the mass density (ρ_m_) of the film is related to the θ_c_, determined by the following equation (1)^20^:

1 $${\theta }_{c}^{2}=(\frac{{e}^{2}{\lambda }^{2}}{\pi m{c}^{2}})\times (\frac{{N}_{A}Z}{A}){\rho }_{m}$$

where λ is the wavelength of X-ray, N_A_ is the Avogadro constant, Z is represented by the mean number of electrons per atom, and A is the mean atomic mass. The critical angle (θ_c_) of the a-STO film increases from the as-deposition to 300 °C. The thickness of s-STO films monotonously decreases from 5.27 ± 0.01 to 5.10 ± 0.06 nm, and the density monotonously increases from 5.90 ± 0.22 to 6.24 ± 0.10 g/cm^3^, as shown in Table 1 (see Figure S8 and Table S1).

**Table 1:** The properties of a-STO films with different annealing processes.

Annealing temperature (°C)	Density (g/cm^3^)	Thickness (nm)	Roughness (nm)
As-deposition	5.90 ± 0.22	5.27 ± 0.01	0.95 ± 0.04
250	6.19 ± 0.05	5.11 ± 0.04	1.05 ± 0.18
300	6.24 ± 0.10	5.10 ± 0.06	1.02 ± 0.20

**Figure 2:**
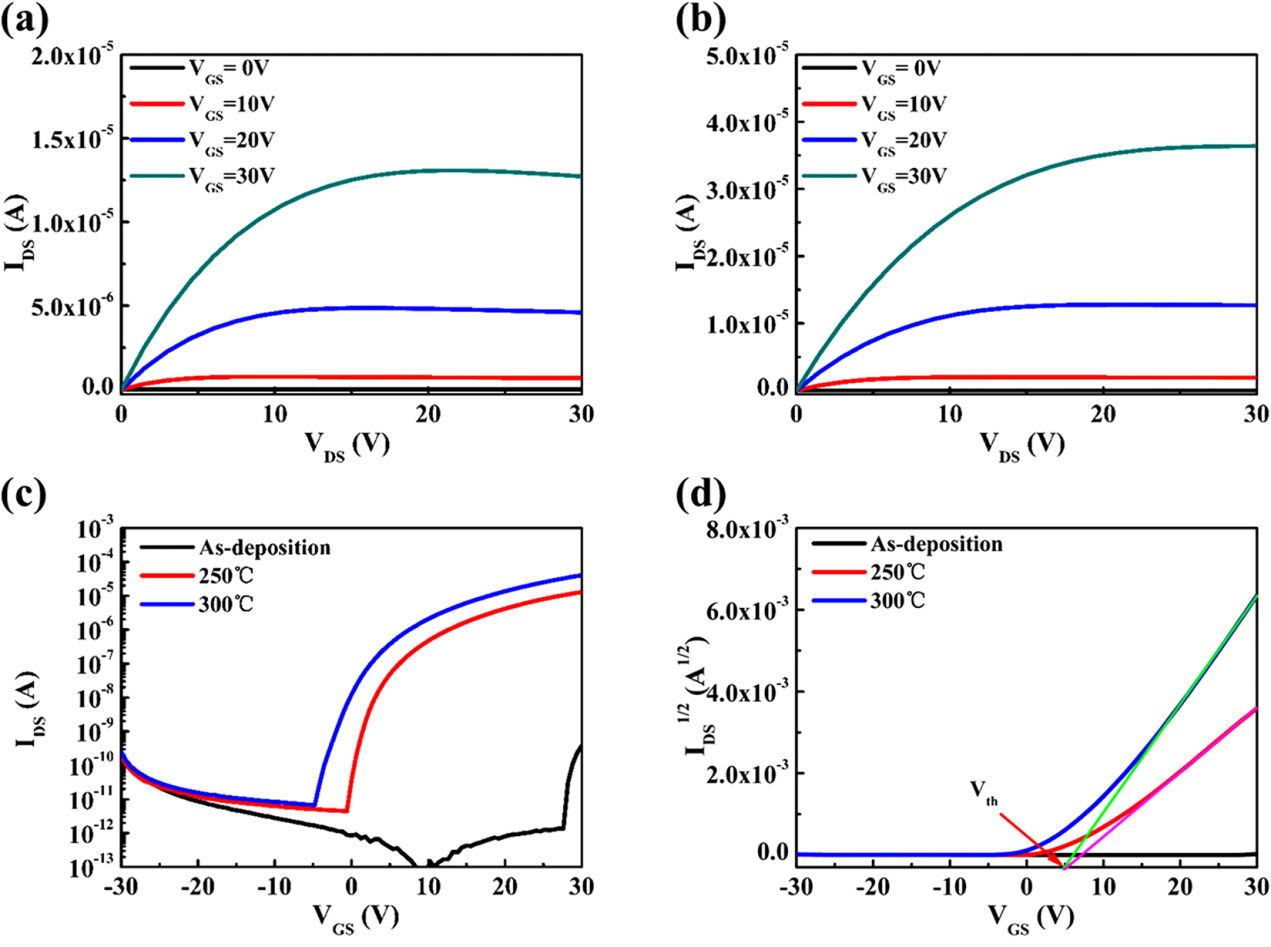
Output characteristic curves (I_DS_ − V_DS_) of the a-STO TFTs annealed at (**a**) 250 °C and (**b**) 300 °C. (**c**) Transfer characteristic curves (I_DS_ − V_GS_) of a-STO TFTs with different annealing temperatures. (**d**) Dependence of the threshold voltage on the annealing temperature of the a-STO TFTs.

The electrical characteristics of a-STO TFTs were investigated using an Agilent 4155 C semiconductor parameter analyzer in the dark air ambient. The output characteristics of TFTs were tested in identical condition. The drain voltage (V_DS_) was swept from 0 to 30 V at a certain gate voltage (V_GS_). During the testing, the V_GS_ was set to 0, 10, 20, and 30 V. Figures 2(a,b) show the output characteristic curves (I_DS_ − V_DS_) of the a-STO TFTs devices annealed at 250 °C and 300 °C in air ambient, respectively. It can be observed that a clear pinch-off of drain current in the channel is seen at zero gate voltage (V_GS_ = 0 V), which is in agreement with a standard theory for the enhanced mode operation of n-type metal oxide semiconductor field-effect transistor (MOFET). It is noteworthy that the drain current of device annealed at 250 °C did not saturate and showed a slight decrease when high gate voltages were applied. This situation is attributed to negative differential resistance effect (NDR effect)^21^, which may be explained that the few stored charges at the Mo/a-STO film interface degrades the current through the junction and the high contact resistance is obtained. The transfer characteristics of the a-STO TFTs were investigated in the saturation regime. A series of tests were carried out in which the gate voltage was swept from −30 to +30 V at a fixed drain voltage of 30.1 V, as shown in Fig. 2(c). And Fig. 2(d) shows the threshold voltage of a-STO TFTs extracted from Fig. 2(c). The low drain current (~10^−10^A) of the as-deposited device was obtained and hardly do exhibit switching characteristics. The turn-on voltage shifted in the negative direction and the on-state current increased as the increase of annealing temperature. The parameters of devices, including the saturation mobility, the threshold voltage, on/off current ratio and sub-threshold swing, were extracted from the transfer characteristic curves, as seen in Table 2. Compared with the device annealed at 250 °C, the a-STO TFTs, annealed at 300 °C, presented relatively a high performance with saturated mobility (µ_sat_) of 6.78 ± 0.14 cm^2^/Vs, a low threshold voltage of 3.41 ± 0.51 V, a on/off current ratio of (5.99 ± 0.49) × 10^6^ and a sub-threshold swing of 0.82 ± 0.15 V/decade (see Table S2).

**Table 2:** Comparison of the various parameters including μ_sat_, V_th_, I_on_/I_off_ ratio and SS for a-STO TFTs annealed at different conditions.

Annealing temperature (°C)	μ_sat_ (cm^2^/Vs)	V_th_ (V)	I_on_/I_off_ (×10^6^)	SS (V/decade)
As-deposition	—	—		—
250	5.24 ± 0.55	4.02 ± 1.77	6.17 ± 0.91	0.70 ± 0.23
300	6.78 ± 0.14	3.41 ± 0.51	5.99 ± 0.49	0.82 ± 0.15

**Figure 3:**
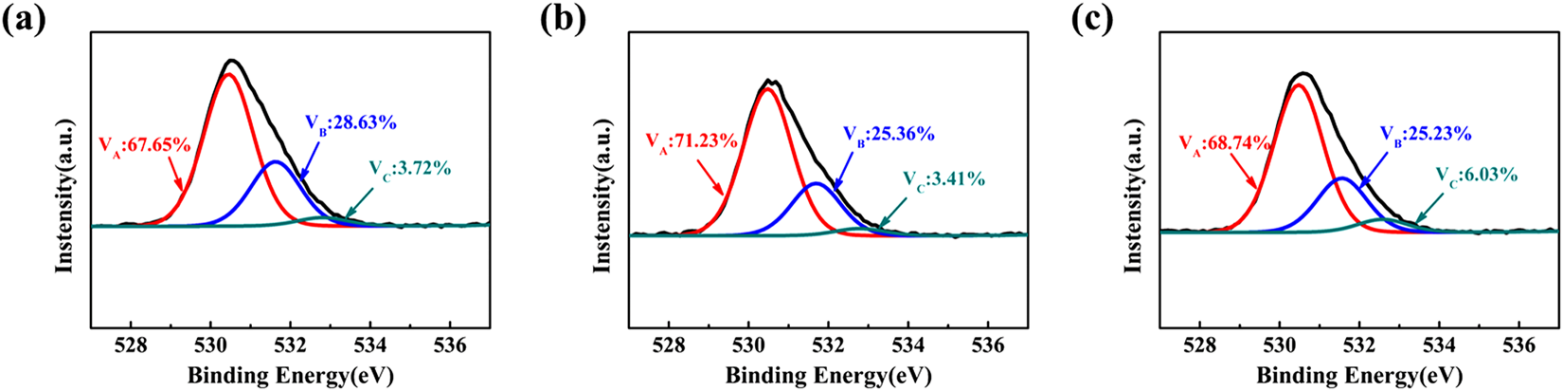
The O 1 s region of XPS spectra for a-STO films with different pre-annealing temperature: (**a**) As-deposition; (**b**) 250 °C; (**c**) 300 °C, respectively.

Then the oxygen vacancy content of the channel region of device was investigated using X-ray photoelectron spectroscopy (XPS), as shown in Fig. 3. The experimental O 1 s peak of each sample was fitted by Gaussian distribution, which could be divided into three Gaussian components centered at 530.49 eV (O_A_), 531.60 eV (O_B_) and 532.76 eV (O_C_), respectively. The peak centers at 530.49 eV and 531.11 eV could be assigned to oxygen ions combined with Sn and Si ions and oxygen vacancies of a-STO film, respectively. The feature at 531.42 eV was associated with the adsorbed oxygen. The O_C_ area proportion (O_C_/(O_A_ + O_B_ + O_C_)) represents the relative quantity of the adsorbed oxygen. The O_C_ content of samples is 3.72% (as-deposition), 3.41% (250 °C and 6.25% (300 °C). Interestingly, it implies that the oxygen-rich area could be formed at the a-STO film surface after it was annealed 300 °C in air.

It is well known that the charge carriers of the oxide semiconductors are related to the oxygen vacancy according to the following equation (2)^22^:

2 $${O}_{O}^{X}=\frac{1}{2}{O}_{2(g)}+{V}_{O}^{\ast \ast }+2{e}^{-}$$

However, the V_B_ content of a-STO film annealed at 250 °C was 25.36% which was close to the V_B_ content (25.23%) of a-STO film annealed at 300 °C. Thus, it is demonstrated that the charge carriers content and resistivity of two a-STO films are similar. But the transfer curve of a-STO TFT annealed at 250 °C did distinguish from the device annealed at 300 °C in aforementioned Fig. 3(c). The reason for the variation of performance is possibly attributed to the contact resistance.

**Figure 4:**
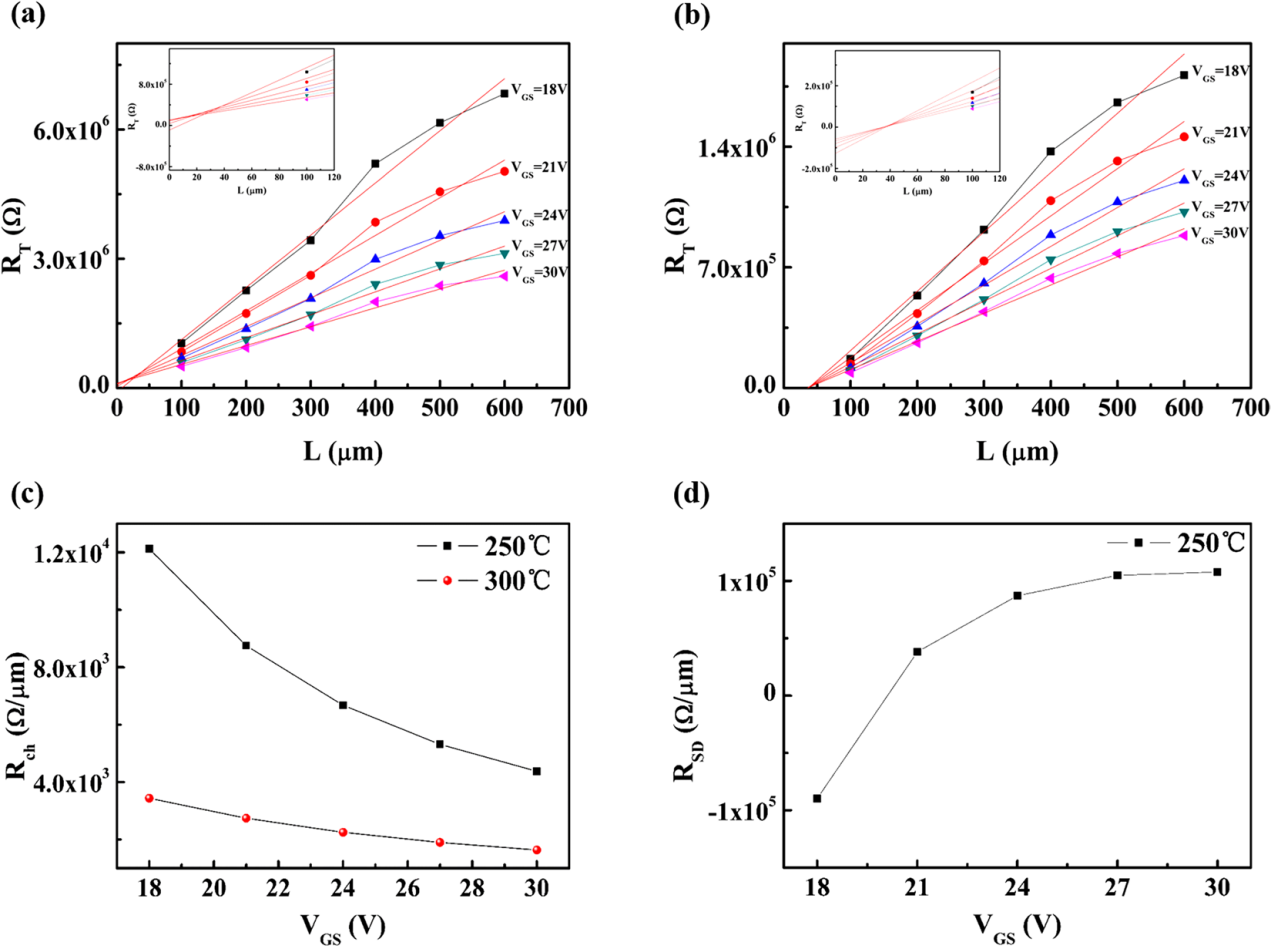
Plot of total resistance (R_tot_) versus channel length (L) for a-STO TFTs annealed at different temperatures: (**a**) 250 °C and (**b**) 300 °C. Plot of R_ch_ (**c**) and R_SD_ (**d**) at source/drain contacts extracted from (a,b) as a function of gate voltage (V_GS_), respectively.

Therefore, to verify above hypothesis, the contact resistance (R_SD_) of a-STO TFTs annealed at 250 °C and 300 °C was investigated using transmission line method (TLM), as shown in Fig. 4(a,b), respectively. The extraction of R_ch_ and R_SD_ is based on the following equation (3) in the linear regime^23^:

3 $${R}_{tot}={R}_{ch}L+{R}_{SD}=\frac{L-{\rm{\Delta }}L}{W{C}_{i}({V}_{GS}-{V}_{T}){\mu }_{eff}}+{R}_{SD}$$

where R_tot_ is the total resistance, R_ch_ is the channel resistance per unit channel length, R_SD_ is the series resistance at the source/drain contacts, the C_i_ is the capacitance per unit area, μ_eff_ is intrinsic field-effect mobility, L is the physical channel length, and ∆L is the difference between the effective channel length and the physical channel length (L) at the source/drain contact, respectively. In our experiment, S/D electrodes have a fixed channel width (W = 600um), but for channel length, it is set up to 100, 200, 300, 400, 500, and 600um. From the linear plot of R_tot_ versus L, R_ch_ and R_SD_ can be obtained from the slope and the intercept of the Y-axis (L = 0), respectively. Usually, the plot of R_tot_ versus L has an intersection point giving the resistance limit (R_0_) value (corresponding to the bulk resistance of channel layer and the contact resistance between channel layer and the source/drain metal) and ∆L value, respectively. But this intersection point could not be found in the TFTs device annealed at 250 °C. It is indicated that there is no highly doped ohmic (n^+^) layer between a-STO film and Mo in a-STO TFTs^24^. However, the intersection point could be found at the annealing temperature of 300 °C, which implied that the ohmic contact was formed at the Mo/a-STO interface. The R_SD_ value was 5.83 × 10^3^Ω, and the ∆L value was 38.45 μm. Commonly, the contact resistance is mainly affected by the interface properties between the channel and S/D metal electrode, including the barrier height, the parasitic capacitance, or the possible oxidation layer. In this paper, the effect of the parasitic capacitance can be excluded because the overlapping region of Mo and a-STO film is identical.

Accordingly, Fig. 4(c,d) show the plot of R_ch_ and R_SD_ of a-STO TFTs extracted from Fig. 4(a,b) as a function of gate voltage (V_GS_), respectively. Compared with slight decrease in R_ch_ of the device annealed at 300 °C, the R_ch_ of the device annealed at 250 °C reduced to the same order of magnitude as the V_GS_ increased. Noticeably, the R_SD_ value of the device annealed at 250 °C was the unreasonable negative value when the L was 0 μm. Then the R_SD_ increased with increasing the V_GS_ value, which could reasonably explain the reduced phenomenon of drain current in high V_DS_ region. And the R_SD_ value tended to be a constant in high V_GS_ region, implying the state of the Mo/a-STO film was close to ohmic contact. It is an artifact error for the negative value of R_SD_ (L = 0 μm) at the annealing temperature of 250 °C, regardless of L variation with respect to the V_GS_. For the ohmic contact of the device annealed at 300 °C, the L variation is negligible, so that this problem was not happened^25^.

**Figure 5:**
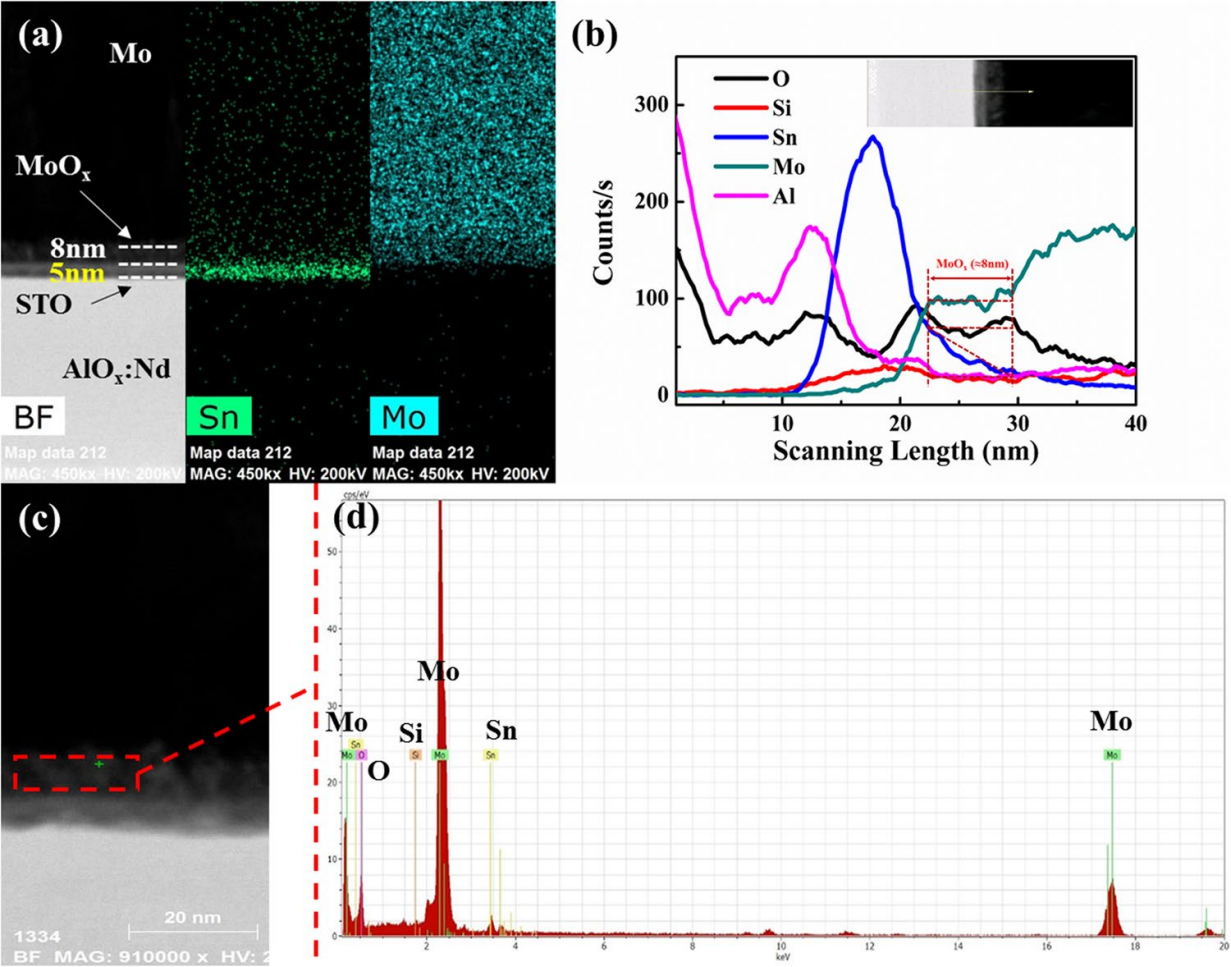
(**a**) Cross-sectional HR-TEM image of a-STO TFT annealed at 300 °C and the element distribution maps of Mo and Sn. (**b**) EDS line scan profiles of cross-sectional a-STO TFT. (**c**) HR-TEM image of the a-STO TFT. (**d**) EDS point scan profiles from (**c**).

For the best performance of a-STO TFT annealed at 300 °C, a focused ion beam transmission electron microscope (FIB-TEM) is utilized to analyze the interface of Mo and a-STO film. Figure 5(a) shows the high-resolution cross-sectional transmission electron microscopy (HR-TEM) image of a-STO TFT and the element distribution maps of Mo and Sn. It is noteworthy that there is a new interlayer between Mo and a-STO film. Meanwhile, the results of energy dispersive spectrometer (EDS) line scan profiles of cross-sectional a-STO TFT and EDS point scan profiles of the new interlayer are shown in Fig. 5(b,d), respectively. It is obvious to see the length of the flat count curve of Mo increases is about 8 nm, and the content of oxygen at the interface is higher than the internal of a-STO film, which implies that the rich-oxygen region is formed in the a-STO film surface. From the data of Fig. 5(b), the area of Mo, Sn and O elements of the new interlayer (scanning length from 22 nm to 30 nm) are approximately calculated with the data of Al element as the baseline. The atomic proportion of O, Mo and Sn elements is 1∶1.51∶ 0.53. The Sn element is found in the new interlayer, and Si element is negligible because the peak signal is very weak, as shown in Fig. 5(d). Thus, the new self-formed interlayer should be the MoO_x_ layer with few Sn atoms. The self-formed interlayer is attributed to the following reasons: 1) The oxygen-rich region at the surface of the a-STO film is formed by pre-annealing treatment. 2) Due to the bond-dissociation energy (BDE) of Mo^+^-O (488.2KJ/mol), Sn^+^-O (281KJ/mol) and Si^+^-O (478KJ/mol), Mo atoms could grab oxygen from the a-STO film surface and replace partial Sn atoms^26^. 3) The replaced Sn atoms diffuse into the new interface layer. Meanwhile, compared with the as-deposition (see Figure S1), the new interlayer, acting as diffusion barrier, can block Mo atoms from diffusing into a-STO film and avoid the deterioration of a-STO TFT performance.

**Figure 6:**
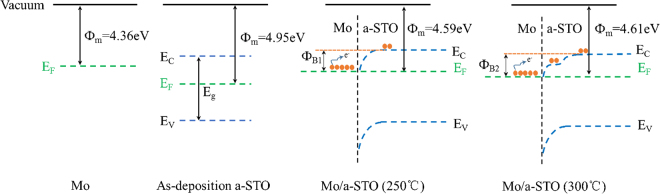
The energy band diagram of a-STO film contacted with Mo.

The on-state current of a-STO TFT nearly increased by an order of magnitude from 250 °C to 300 °C. This might be caused by the low R_ch_ and the reduced electronic transmission barrier in the Mo/a-STO interface. The work function of Mo film (100 nm), 5 nm thick a-STO films (as-deposition, 250 °C and 300 °C) were measured using Kelvin probe. Figure 6 shows the energy band diagram of a-STO film contacted with Mo. The work function of Mo films is 4.36 eV, and a-STO has a work function of 4.95 eV (as-deposition), 4.59 eV (250 °C) and 4.61 eV (300 °C). The work function of a-STO film decreased as the increase of thermal annealing temperature. After the a-STO films annealed at 250 °C and 300 °C, both of a-STO films matched with Mo had nearly the same barrier height. However, the obtained output current of device pre-annealed at 300 °C was larger than that of device with a pretreated temperature of the 250 °C. This is attributed to the new interlayer that results in the formation of stepwise internal transport barrier, which is beneficial for electrons to inject from Mo into a-STO film. Herein, introducing an intermediate energy-level modification layer between the electrode and the channel layer could improve the contact property and reduce signal delayer.

## Conclusion

In summary, a viable and effective solution was developed for improving the contact property of a-STO TFTs. The ~8 nm thick self-formed metal-oxide interlayer caused by the oxygen-rich of a-STO film surface during pre-annealing process. It could facilitate the formation of ohmic contact at the Mo/a-STO interface with the pre-annealing temperature of 300 °C, showing a R_SD_ of 5.83 × 10^3^Ω. The new interlayer can improve the contact quality and decrease electron injection barrier height without inserting an extra barrier layer or a highly doped ohmic (n^+^) layer between semiconductor and the source/drain metal. The method of interface self-modification is potential for reducing RC delay in the AOS-TFTs backplane in the future.

## Methods

### Device Fabrication

Silicon-Tin-Oxide (STO) films were deposited from STO target (SiO_2_:SnO_2_ = 5:95 wt%) by Radio frequency (RF) Magnetron sputtering (Kurt J. Lesker). The STO films were deposited at the power of 80 W, the pressure of 2mtorr and the Argon/oxygen flow ratio of 7.4/0.74sccm. The device configuration of TFT is composed of inverted staggered type. A 300 nm thick Al-Nd alloy (3 wt% of Nd) as gate metal was deposited by direct current (DC) magnetron sputtering and its patterns were defined by photolithography. Subsequently, the film was anodized to form a 200 nm layer of AlO_x_-Nd on the surface in an electrolyte consisting of 3.68 wt% ammonium tartrate solution and ethylene glycol. Afterwards, a 5 nm thick STO thin film was deposited on the anodic oxide film by RF sputtering at room temperature. Before the S/D electrodes deposition, it was annealed at 250 °C and 300 °C in air ambient for 0.5 h, respectively. For the S/D electrodes, 100 nm thick Mo metal was sputtered to form the S/D electrodes through metal shadow mask, defining a channel width/length of 300/300 μm. No passivation layer was deposited after the a-STO TFT fabrication.

### Measurement and Characterization

The thickness of a-STO film was measured by X-ray reflectivity (XRR, Empyrean, PANalytical). The electrical characteristics of TFTs were investigated by the semiconductor parameter analyzer (Agilent, 4155 C) in the dark air ambient. The oxygen vacancy content of a-STO films were analyzed by X-ray photoelectron spectroscopy (Thermo, ESCALAB 250 Xi). The cross-sectional microstructure of TFT was analyzed by HRTEM.

## Electronic supplementary material

Supplementary Information
