# Supplementary material for: Induced nano-scale self-formed metal-oxide interlayer in amorphous silicon tin oxide thin film transistors
Source: Sci Rep. 2018 Mar 7;8:4160. doi: 10.1038/s41598-018-22602-4 (PMC5841428; doi:10.1038/s41598-018-22602-4)
Supplement: Supplementary file 1 — Supplementary Information [file 41598_2018_22602_MOESM1_ESM.pdf]

Induced nano-scale self-formed metal-oxide interlayer in amorphous silicon tin oxide thin film transistors

Xianzhe Liu[1], Hua Xu[1], Honglong Ning[1,*], Kuankuan Lu[1], Hongke Zhang[1], Xiaochen Zhang[1], Rihui Yao[1,*], Zhiqiang Fang[1], Xubing Lu[2], Junbiao Peng[1]

[1] Institute of Polymer Optoelectronic Materials and Devices, State Key Laboratory of Luminescent Materials and Devices, Department of Materials Science and Engineering School, South China University of Technology, Guangzhou 510640, China

[2] Institute for Advanced Materials and Guangdong Provincial Key Laboratory of Quantum Engineering and Quantum Materials, South China Normal University, Guangzhou 510006, China

E-mail: ninghl@scut.edu.cn; yaorihui@scut.edu.cn

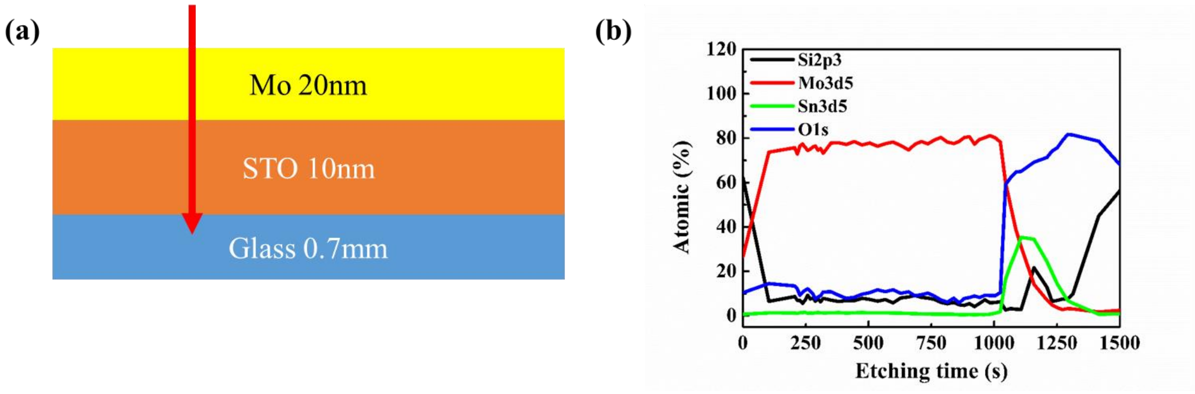

Figure S1. (a) the sample of as-deposited a-STO film matched with Mo, (b) the spectra of XPS depth profiles obtained from the Mo layer to the glass corresponding to Figure S1 (a).

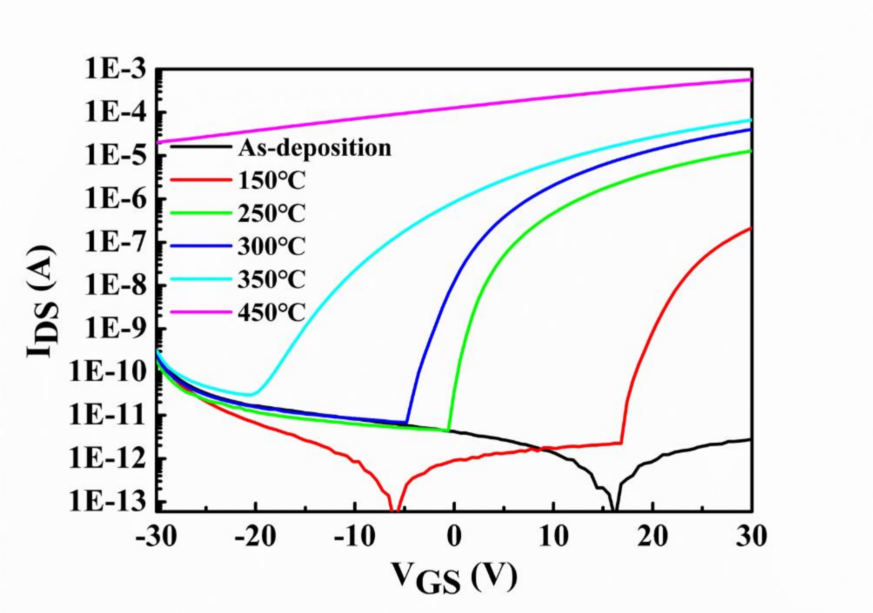

Figure S2. Transfer characteristic curves (I_{DS}-V_{GS}) of a-STO TFTs with different annealing temperatures.

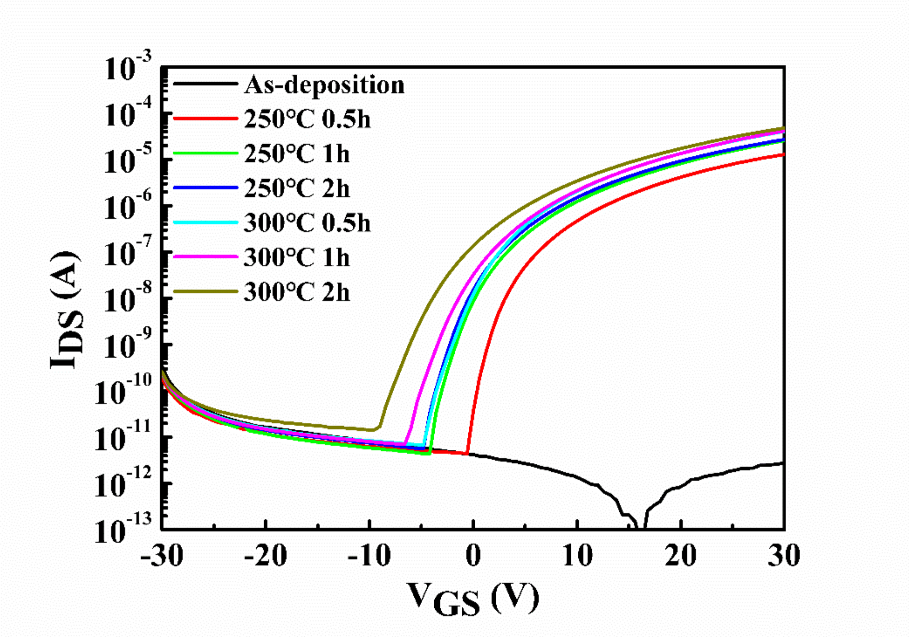

Figure S3. Transfer characteristic curves (I_{DS}-V_{GS}) of a-STO TFTs with different annealing temperatures in different annealing times.

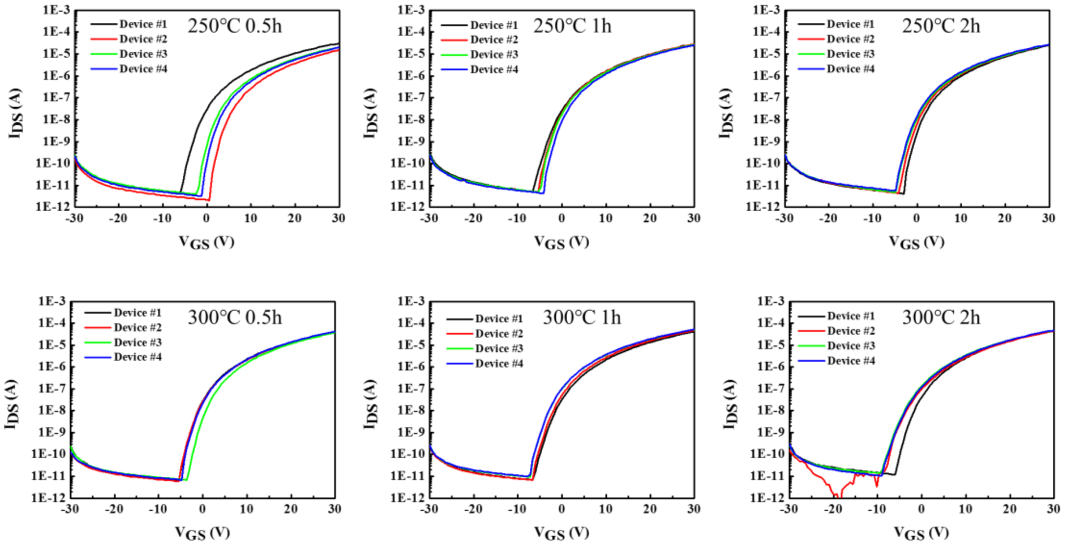

Figure S4. Transfer characteristic curves (I_{DS}-V_{GS}) of a-STO TFTs with different annealing times in 250C and 300C.

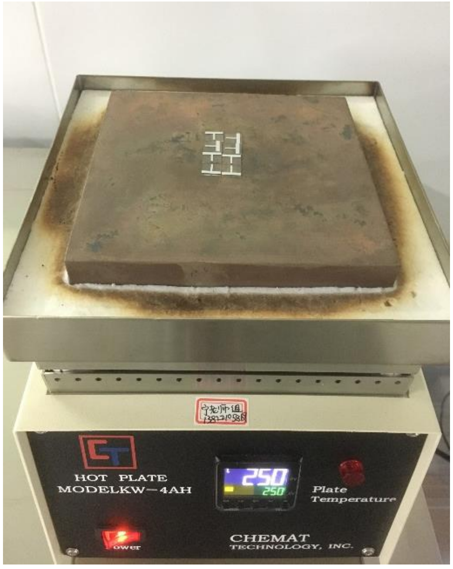

Figure S5. The device annealed on the hot plate.

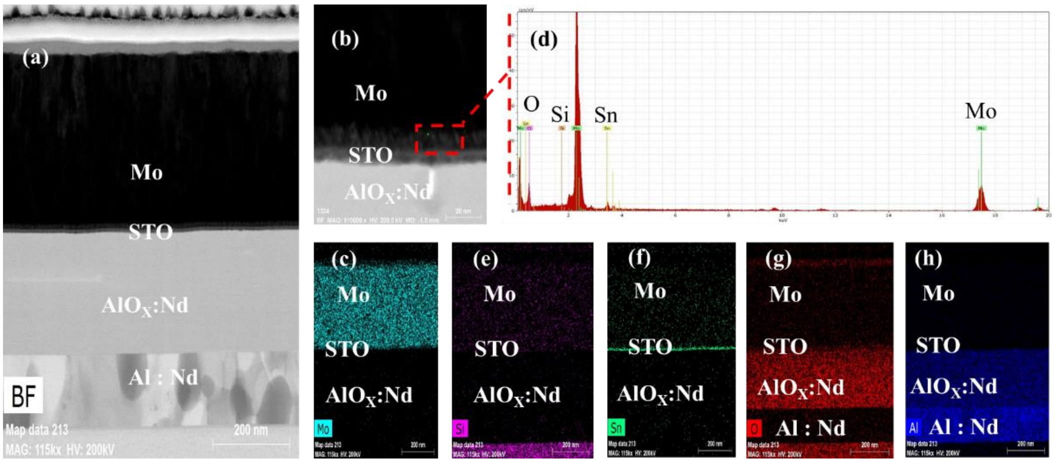

Figure S6. (a) Cross-sectional HR-TEM image of a-STO TFT annealed at 300℃. (b) partial cross-sectional HR-TEM image of a-STO TFT and the element distribution maps of Mo (c), Si (e), Sn (f), O (g) and Al (h). (d) EDS point scan profiles from (b).

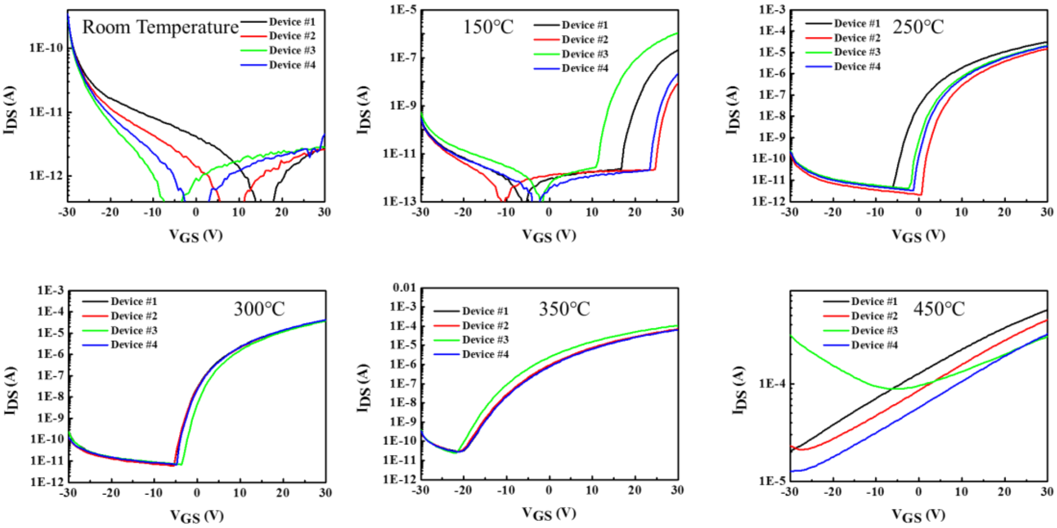

Figure S7. The transfer curves of devices annealed at different pre-annealing temperatures.

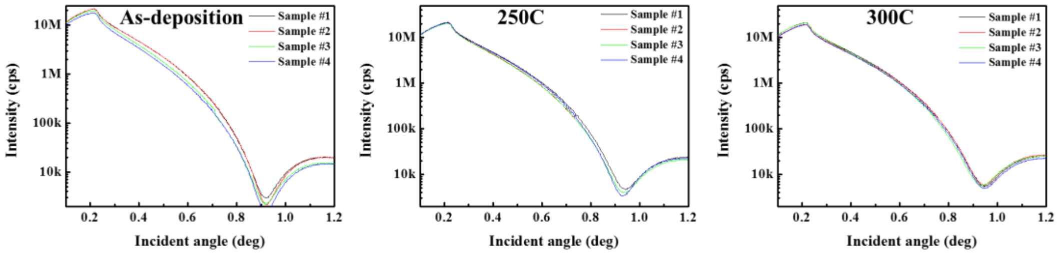

Figure S8. X-ray reflectivity (XRR) curves for different annealing processed a-STO films (as-deposition, annealed at 250℃ and 300℃ in air ambient).

Table S1. The properties of a-STO films at different annealing temperatures. Every four samples were tested in every annealing temperature.

Temperature	Density(g/cm^3)	Thickness(nm)	Roughness(nm)
RT 1	6.094	5.266	0.925
RT 2	6.074	5.266	0.911
RT 3	5.711	5.27	0.967
RT 4	5.709	5.281	0.989
**RT Average value**	**5.897**	**5.271**	**0.948**
250C 1	6.123	5.168	0.786
250C 2	6.202	5.092	1.165
250C 3	6.186	5.079	1.147
250C 4	6.237	5.113	1.113
**250C Average value**	**6.187**	**5.113**	**1.053**
300C 1	6.379	5.033	1.074
300C 2	6.161	5.147	0.721
300C 3	6.209	5.081	1.141
300C 4	6.203	5.15	1.125
**300C Average value**	**6.238**	**5.103**	**1.015**

Table S2. Comparison of the various parameters including μ_{sat}, V_{th}, I_{on}/I_{off} ratio and SS for a-STO TFTs annealed at different conditions.

Annealing temperature (℃)	μ_{sat} (cm^2/Vs)	V_{th} (V)	I_{on}/I_{off} ($\times 10^6$)	SS (V/decade)
As-deposition	-	-	-	-
150	0.50±0.39	20.68±5.16	(12.72±18.92)	1.152±0.21
250	5.24±0.55	4.02±1.77	(6.17±0.91)	0.70±0.23
300	6.78±0.14	3.41±0.51	(5.99±0.49)	0.82±0.15
350	8.26±0.14	-3.23±4.13	(2.86±1.07)	2.86±0.18
450	-	-	-	-
